# Evaluation of Continuous UVC Treatments and its Combination with UHPH on Spores of *Bacillus subtilis* in Whole and Skim Milk

**DOI:** 10.3390/foods8110539

**Published:** 2019-11-02

**Authors:** María Martinez-Garcia, Jezer N. Sauceda-Gálvez, Idoia Codina-Torrella, Mª Manuela Hernández-Herrero, Ramón Gervilla, Artur X. Roig-Sagués

**Affiliations:** 1 Centre d’Innovació, Recerca i Transfèrencia en Tecnologia dels Aliments (CIRTTA), XaRTA, TECNIO-CERTA, MALTA-Consolider Team, Departament de Ciència Animal i dels Aliments, Facultat de Veterinària, Universitat Autònoma de Barcelona, 08193 Bellaterra, Spain; maria.martinez.garcia@uab.cat (M.M.-G.); idoia.codina@uab.cat (I.C.-T.); manuela.hernandez@uab.cat (M.M.H.-H.); 2 SPTA-Servei Planta Tecnologia Aliments, Universitat Autònoma de Barcelona, c/ de l‘Hospital S/N, 08193 Bellaterra (Barcelona), Spain; ramon.gervilla@uab.cat

**Keywords:** UVC, UHPH, milk, *Bacillus subtilis*

## Abstract

The aim of this study was to evaluate the effectiveness of different UVC treatments, alone or in combination with ultra-high pressure homogenization (UHPH) on *Bacillus subtilis* spores in milk. Spores of *B. subtilis* (CECT4002) were inoculated in whole and skim milk to an initial concentration about 6 log CFU/mL. Milk was subjected to different ultraviolet radiation treatments at 254 nm (UVC) using a concentric tubular reactor in a dose ranging from 10 to 160 J/mL. Different number of passes were used to adjust the final dose received by the matrix. In general, increasing the number of passes (defined as number of entries to the tunnel-NET) increased the inactivation of spores of *B. subtilis*. The best lethality results (above 4 Log CFU/mL) were obtained by applying doses from 100 J/mL with several NET. When the same doses were achieved with a single pass lethality in most cases did not exceed 1 log CFU/mL. Increasing the NET also increased the likelihood for the spores to remain longer in the effective distance from the UVC source, estimated as 0.02 mm for whole milk and 0.06 mm for skim milk. Combination of UHPH and UVC did not clearly increase the efficiency of a single UVC treatment, and a lower lethality was even observed in some cases. UHPH treatments increased the turbidity and absorption coefficient (254 nm) of both whole and skim milk.

## 1. Introduction

Short-wave ultraviolet radiation (UVC) is a non-thermal technology proposed as an alternative to heat pasteurization that is being investigated nowadays for the reduction of microorganisms in liquid foods that may lead to spoilage and potential health risks for the consumers [1]. The application of UVC using reactors allows processing liquid foods in a continuous regime which increased the interest on this technology because it is easy to use, has a lower cost and offers a better preservation of the nutritional content and quality aspects of foods [2,3,4,5]. UVC is lethal for most of microorganisms through the formation of pyrimidine dimers which blocks DNA duplication leading to cell death [6,7]. Moreover, unlike other non-thermal emerging technologies, UVC is capable of inactivating bacterial spores [8,9].

The microbiocidal effect of UVC has been tested in different foodstuffs, such as milk, demonstrating an effectiveness similar to thermal pasteurization [2,10,11,12,13,14,15,16,17,18,19,20,21,22]. However, milk presents the limiting factor of its high absorption coefficient at 254 nm (α(254)) which reduces the possibility for UVC radiation to penetrate through the matrix and reach those areas farther from the UVC source with the same intensity, underexposing some of the present microorganisms, and consequently, reducing the effectiveness of the UVC treatment [23,24,25,26].

In order to increase the microbiocidal efficiency of UVC when a continuous flow reactor is used, both the UVC radiation dose and the flow pattern (laminar flow or turbulent flow) must be correctly adjusted to reduce the distance from the UVC source and the microorganisms to make them access the area were the UVC radiation is more efficient inside the reactor [4]. Two basic designs of continuous flow UVC reactors are the most frequently described in the literature to overcome these inconveniences. Some are based on increasing the turbulence generated inside the system and consequently the probability that all parts of the matrix could get into close contact with the UVC source. The conditions for a turbulent flow are described mathematically by the Reynolds number (Re), a minimum Re of 2100 should be achieved to ensure a sufficient turbulence to guarantee a more homogeneous distribution of the residence times inside the reactor [24,27]. The other proposed strategy is to reduce as much as possible the gap between the UVC source (lamp) and the farthest particle of the matrix, reducing the path length through creating a thin film around the UVC source. However, in this system, the flow of the liquid is laminar, and that represents an inconvenience to treat matrices with high α(254). One option to overcome this inconvenience is to pass the matrix several times throughout the reactor or several connected reactors, forcing a mixing effect between each pass that increases the efficiency.

A different strategy to increase the UVC treatments efficiency would be to combine them with another emerging non-thermal technology, such as ultra-high pressure homogenization (UHPH) seeking for a synergic o additive effect following the “hurdle” principle. UHPH has been proposed as a good alternative to thermal pasteurization as it reduces the load of vegetative cells of pathogen and spoilage microorganisms significantly while sparing most of the organoleptic and physicochemical characteristics of raw milk [28,29,30]. However, its effect on bacterial spores is much more limited [31,32]. Both strategies have been recently tested on *Bacillus subtilis* inoculated into phosphate-buffer saline solution (PBS), increasing the efficiency compared with both UVC and UHPH single treatments [33]. A complementary effect between these technologies was also reported, as UVC was able to inactivate microorganisms that were resistant to UHPH and vice versa.

The aim of this study was to evaluate the effectiveness of UVC treatments applied in different ways, alone or in combination with UHPH on *Bacillus subtilis* inoculated in whole and skim milk, and exploring the potential of these technologies to obtain commercial sterile products. Mathematical models were used to determine the importance of the treatment variables on their efficacy.

## 2. Materials and Methods 

### 2.1. Preparation of the Spore Suspension of B. Subtilis

The strain used in this study was *Bacillus subtilis* CECT4002, supplied by the Spanish Type Culture Collection (CECT, University of Valencia, Valencia, Spain). A modification of the UNE EN ISO 13704:2002 procedure [34] was used to obtain the spores. Briefly, the lyophilized culture was rehydrated in 10 mL of glucose and tryptone broth (TGB: 2.5 g of yeast extract (Oxoid, Basingstoke, UK), 5 g of tryptone (Oxoid), 1 g of glucose (Sigma-Aldrich, St. Louis, USA), in 1 L of distilled water, pH adjusted to 7.2), and incubated for 24 h at 30 °C. Two mL of this culture was transferred to Roux bottles containing yeast extract agar (MYA: 10 g of meat extract (Oxoid), 2 g of yeast extract (Oxoid), 15 g of agar (Oxoid) and 0.04 g of MnSO_4_·H_2_O (Merck, Darmstadt, Germany) in 1 L of distilled water), which were incubated at 30 °C for up to 30 days. The formed spores were collected by adding 20 mL of sterile distilled water to the Roux bottles and scraping the surface with a Digralsky stick. Spore suspensions were pooled and washed four times in 15 mL cold sterile water by centrifugation at 10,000× *g* for 20 min at 4 °C using a Sigma 4K15 centrifuge (Sigma Laborzentrifugen GmbH, Osterode am Harz, Germany). The resulting sediment was then suspended in 30 mL of sterile distilled water and subjected to a heat treatment at 75 °C for 10 min to ensure the inactivation of vegetative cells. The resulting spore suspension was stored at 4 °C until use. One mL of the spore suspension was used to inoculate 1 L of milk to guarantee a minimum initial load close to 10^6^ CFU/mL.

### 2.2. Types of Milk Used 

Two types of locally purchased UHT cow milk were used to carry out this study: whole milk (3.5% Fat) and skim milk (<0.2% Fat). The characteristics of the matrices that may influence the efficacy of the treatments were determined with the following methods.

#### 2.2.1. Absorption Coefficient at 254 nm (α(254)) 

It was determined using a Nanophotometer Pearl model spectrophotometer (IMPLEN GmbH, München, Germany) with 1 cm quartz cuvettes (Fisher Scientific, Hanover, IL, USA). Sample was diluted 1000 fold with distilled water in order to fit into the reliable reading range of the spectrophotometer.

#### 2.2.2. Turbidity

It was measured with a portable EPA 2100Q turbidimeter (HACH, Hospitalet de Llobregat, Spain). Sample was diluted 1000 fold with distilled water in order to fit into the reliable reading range of the turbidimeter. Results were expressed as Nephelometric Turbidity Units (NTU).

### 2.3. Application of UVC Radiation Treatments

#### 2.3.1. UVC Reactor Features

**Figure 1:**
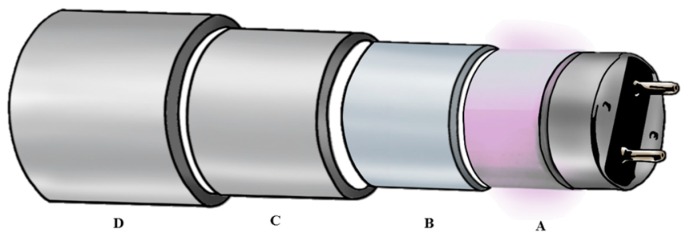
Structure of the UVC reactor used in this survey. A: UVC lamp; B: quartz protection glass; C and D stainless steel tubes. Section A–B: air circulation; section B–C: food matrix circulation area; section C–D: water cooling system.

A UVC reactor (UV-Therm, Ypsicon S.L., Barcelona, Spain; European Patent EP 2965766-A1) was used in this survey. The reactor was fed by a Flowmaster FMT300 peristaltic pump (ISMATEC Lab. GmbH, Wertheim-Mondfeld, Germany) through a silicone pipe. It has a capacity of 673 mL of total volume and consists of two low pressure mercury UVC lamps (UV-Consulting Peschl Spain, Castellón, Spain), with a total electrical power of 55 W and an irradiance of 41 mW/cm^2^. Each of the lamps is protected by a 2 mm thick quartz tube (UV-Consulting Peschl, Geldo, Spain), leaving a radial space to circulate for the food of 1 mm. The internal section of the UVC reactor consists of different concentric cylinders (Figure 1): between sections A and B air circulates to cool the UVC lamp; between sections B and C the food matrix circulates in a layer of 1 mm; externally (between sections C and D) water flows to control the temperature of the treatments. In this survey, the temperature was adjusted to 20 °C.

#### 2.3.2. UVC Treatments

The parameters that defined each UVC treatment were: the UVC energy emitted by the lamp per volume unit (dosimetry); the flow rate at which the sample circulated (expressed as mL/s) and correlated with the revolutions per minute or rpm of the peristaltic pump) that determines the retention time inside the UVC lamp during each pass; and the number of passes through the reactor defined as the number of entries into the reactor or NET. Table 1 shows the combination of these parameters that defined each treatment. The different combinations were divided into three groups depending on the flow rate used (T1, T2 and T3). In each group, the final doses achieved ranged from 20 to 160 J/mL.

#### 2.3.3. Dosimetry Using a Potassium Iodide/Iodate Actinometer

In this study, the UVC energy emitted by the lamp per volume unit at 254 nm was measured by the chemical iodide/iodate actinometer developed and described by Rahn [35] using a solution of potassium iodide that was passed through the UVC reactor. Its absorbance was then measured at 352 nm using a Nanophotometer Pearl spectrophotometer (IMPLEN) after diluting ten times the sample with distilled water. The formula proposed by Linden and Mofidi [36] was used to calculate the power of the UVC reactor lamps:

(1) $$H_{\mathrm{MP}}=\frac{\left({\Delta \mathrm{OD}}_{352}\right)\left(V\right)\left(23,786.4\right)}{\left(I\right)\left(1+0.02\left(T-20.7\right)\right)A}$$

where H_MP_ is the applied UVC dose of the UVC lamp in mJ/cm^2^; ΔOD_352_ ${\Delta \mathrm{OD}}_{352}$ is the difference of the absorbance of the irradiated sample and absorbance of the untreated (white) sample measured at 352 nm; V is the volume of the irradiated sample in L; I is the path length of light passing through the solution in cm (in the reactor used it was 0.1 cm); T is the temperature of the treatment, expressed in °C; A is the area of sample facing the light source (700 cm^2^) and 23,786.4 is the constant (mJ/cm^2^) specific for LMP-UVC lamps.

#### 2.3.4. Determination of the Type of Flow into the Reactor

To determine the type of flow at which the matrix was circulating through the reactor the Reynolds number (Re) was determined according to the formula described by Müller et al. [37]:

(2) Re = d·v·ρη

where *d* is the diameter of the tube (m), v is the velocity (m/s), ρ is the fluid density (kg/m^3^), and η is dynamic viscosity of the fluid (Pa·s). The relative viscosity was obtained using an Ostwald 1293 model viscometer (CIVEQ, Mexico City, Mexico) and density with a hydrometer–aerometer densitometer HYDR-100-001 (Labbox, Vilassar de Dalt, Spain).

According to that, the flow is considered laminar when the value of Re is less than 2100 and turbulent when it is above 4000. Table 1 shows the Re values obtained in the treated samples.

#### 2.3.5. Determination of the Effective Depth of UVC

In liquid matrices of high opacity or turbidity the UVC photons cannot penetrate deeply into the liquid, for this reason, it is necessary to know the depth at which the action of the UVC_254 nm_ radiation is effective. This can be determined by applying the *Lamber-Beer* law by the equation:

(3) $$I=I_{0}\cdot e^{-k\cdot c\cdot d}$$

where I and I_0_ correspond to the intensity of the UVC radiation expressed in mW/cm^2^; I corresponds to the intensity that the matrix receives at a given point, and I_0_ corresponds to the intensity emitted by the reactor. The constant k corresponds to the α(254) of the matrix expressed in cm^−1^; c is the concentration of solutes capable of absorbing UVC of the sample expressed in mol/L, and finally, d is the depth or distance at that must UVC light pass through expressed in cm.

### 2.4. UHPH Treatments

For these experiments, an ultra-high pressure homogenizer Stansted FPG12500 (Stansted Fluid Power Ltd., Essex, United Kingdom), with a flow rate of 15 L/h, was used. The equipment consisted in two intensifiers driven by a hydraulic pump and a pressure valve consisting of a combination of a needle and a zirconium seat, with a cutting angle of 60 ± 0.5° at the needle and 45 ± 0.5° in the seat. Two heat exchangers (Garvía, Barcelona, Spain) were placed before and after the UHPH equipment to control the inlet and outlet temperatures.

Whole and skim milk inoculated with spores of *B. subtilis* were treated at a pressure of 200 MPa in a single stage at an inlet temperature of 60 °C. Then, the samples were subjected to the UVC treatment conditions T1 and T3 (Table 1).

### 2.5. Microbiological Analysis of the Samples and Calculation of the Lethality Achieved

Once treated, samples were collected aseptically in a Telstar PCR Mini-V biosafety cabinet (Telstar, Terrassa, Spain), in sterile containers, and kept refrigerated (4 °C) until analysis. Ten-fold dilutions were made from each sample in phosphate buffered saline solution consisting of 0.24 g of KH_2_PO_4_ in 1 L of distilled water and pH adjusted to 7.4 (PBS, Panreac). Aliquots of each dilution were plated in Petri plates with trypticase soy agar medium enriched with 0.6% yeast extract (TSA-YE, Oxoid). The plates were incubated for 24 h at 37 °C.

The lethality caused by the UVC and/or UHPH treatments on *B. subtilis* was estimated with the formula:

(4) $$\mathrm{Lethality}={\;\mathrm{Log}}_{10}\left(\frac{N_{0}}{N+1}\right)$$

where N_0_ is the initial amount of spores of *B. subtilis* present in the samples before the treatments and N is the number of remaining viable spores after the treatments, both expressed as in CFU/mL.

### 2.6. Determination of the Inactivation Kinetics and Effect of Treatment Variables

Different statistical models obtained from the results obtained with the different matrices after the UVC treatments (T1, T2 and T3) were elaborated based on the lethality as a response variable, and different explanatory or independent variables: (1) Log C, corresponding to the quantity of inoculated microorganisms expressed as log CFU/mL; (2) UVC, the dose of UVC radiation, expressed in J/mL; (3) the treatment flow rate, expressed in mL/s; and (4) the NET, expressed as its absolute value. Five models were elaborated: model 1 presents the effect of the combination of variables of dose and flow rate with a linear adjustment; model 2 presents the influence of the NET value; model 3 also presents the influence of NET but from a quadratic approach; model 4 included the variables of UVC dose, flow rate and NET with a linear adjustment, and finally model 5 is a quadratic adjust using the variables dose, flow rate and NET.

### 2.7. Determination of the UVC Four Decimal Reduction Value (4Duvc)

Survival data of *B. subtilis* spores after the different UVC treatments (T1, T2 and T3) were adjusted to different linear and nonlinear models of survival curves (biphasic, Weibull, Weibull with tail, Double Weibull and biphasic models with shoulder) using the GInaFiT software a freeware add-in for Microsoft^®^ Excel [38]. The suitability of the adjustment was determined by determining the root mean square error (RMSE) value and the coefficient (*R*^2^). The 4D_uvc_ value that is the UVC dose necessary to reduce 4 log CFU/mL of the initial load of the microorganisms tested was determined from the models that show the best adjustment.

### 2.8. Statistical Analysis of the Results

Three independent trials were performed for each experiment and two different samples, from each trial (*n* = 6). Analysis of variance (ANOVA) and Tukey test was used to compare results between treatments and/or matrices. Differences were considered to be significant at *p* < 0.05. This analysis was performed with the R system for statistical computation (R Foundation for Statistical Computing, Vienna, Austria 2014, http://www.R-project.org).

## 3. Results

### 3.1. Lethal effect of UVC Treatments on Bacillus Subtilis Spores

**Figure 2:**
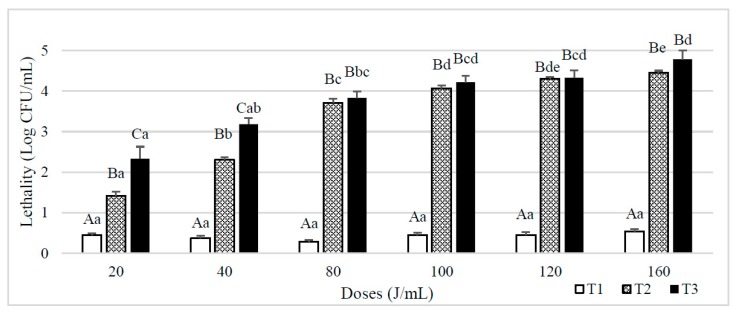
Lethality of *B. subtilis* spores inoculated in whole milk caused by different UVC treatments consisting of different doses (J/mL) and number of entries to the reactor (T1, T2 and T3). Results are expressed as log CFU/mL ± standard deviation. Different lowercase letters in the columns indicate significant differences (*p* < 0.05) between processes with the same flow rate (T1, T2 and T3, see Table 1), but different doses of UVC. Different capital letters indicate significant differences (*p* < 0.05) between processes with different flowrate, but the same dose of UVC.

Figure 2 shows the lethality results obtained on spores *B. subtilis* inoculated in whole milk. When a single pass through the reactor was used (T1), lethality was always below 1 log CFU/mL, and no significant differences (*p* ≥ 0.05) were observed between UVC doses. When the number of passes increased (T2 and T3), there was a significant increase in the lethality value, obtaining a greater reduction in the T3 treatments than in the T2 although differences were reduced when the dose increased. Lethality also increased significantly with the final UVC dose received until achieving a final dose of 80 J/mL. From this dose and above, statistical differences depending on both NET and the final UVC dose were not observed.

**Figure 3:**
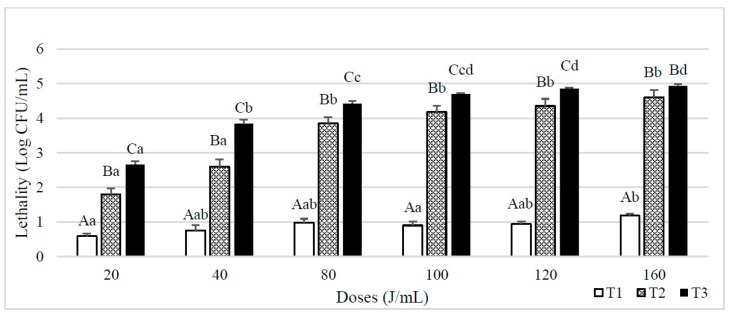
Lethality of *B. subtilis* spores inoculated in skim milk caused by different UVC treatments consisting of different doses (J/mL) and number of entries to the reactor (T1, T2 and T3 Results are expressed as log CFU/mL ± standard deviation. Different lowercase letters in the columns indicate significant differences (*p* < 0.05) between processes with the same flow rate (T1, T2 and T3, see Table 1), but different doses of UVC. Different capital letters indicate significant differences (*p* < 0.05) between processes with different flowrate, but the same dose of UVC.

When skim milk was inoculated with spores of *B. subtilis* (Figure 3), similar results were observed. T1 treatments lethalities were closer to 1 log CFU/mL when the UVC dose was of 80 J/mL and above, being maximum (1.2 log CFU/mL) after a dose of 160 J/mL. In that case, lethalities also increased when higher NET and UVC doses were applied.

Table 2 shows the minimal distance in which the action of UVC radiation is still effective. It was determined at the point where the received dose was 1 mJ/cm^2^. Whole milk had the highest α(254), and in consequence, the distance that UVC radiation can penetrate without interference was shorter. Due to this reduced effective distance, increasing the number of passes (determined by the NET), increased the probability for a *B. subtilis* spore to get into this effective zone, increasing the real UVC received dose and consequently the effectiveness of the treatment.

### 3.2. Lethal Effect of UHPH-UVC Combined Treatments on Bacillus Subtilis Spores

**Figure 4:**
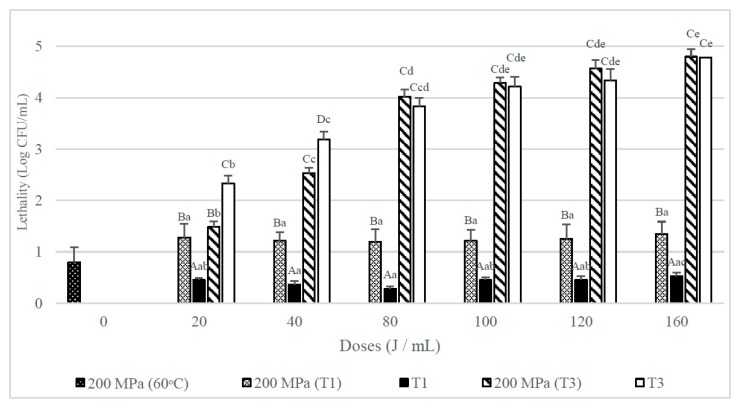
Lethality of *B. subtilis* spores inoculated in whole milk from UHPH treatments at 200 MPa and UVC treatments at different doses (J/mL) and their combination with different number of passes (T1 and T3). Results are expressed as log CFU/mL ± standard deviation. Different lowercase letters in the columns indicate significant differences (*p* < 0.05) between processes with the same flow rate (T1 and T3, see Table 1), but different doses of UVC. Different capital letters indicate significant differences (*p* < 0.05) between processes with different flowrate, but the same UVC dose.

Figure 4 shows that the lethality obtained when whole milk inoculated with spores of *B. subtilis* was submitted to an UHPH treatment at 200 MPa at an inlet temperature of 60 °C was below 1 log CFU/mL. When samples were T1 treated after the UHPH treatment a mean reduction above 1 log CFU/mL was achieved, that was statistically greater than those achieved when only a UVC treatment was used (*p* < 0.05). However, when combining the UHPH treatment with the T3 UVC treatments at the mildest doses (20 and 40 J/mL) the lethality obtained was less than in single UVC (T3) treatments, although at higher doses (80–160 J/mL) no significant differences between the combined and simple treatments were observed.

**Figure 5:**
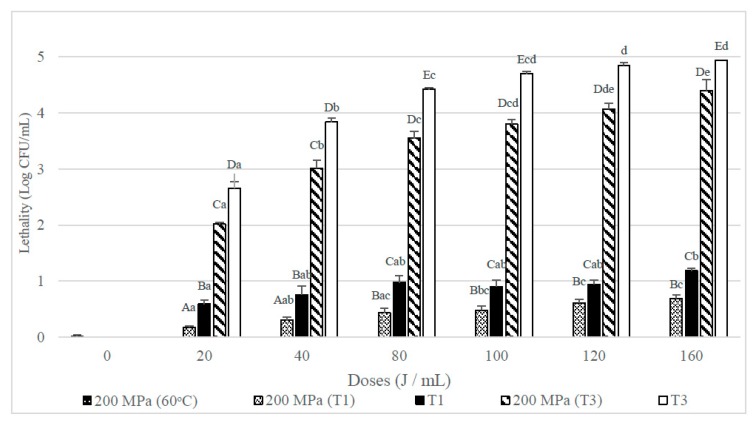
Lethality of *B. subtilis* spores inoculated in skim milk caused by UHPH treatments at 200 MPa and UVC treatments at different doses (J/mL) and their combination with different number of passes (T1 and T3). Results are expressed as log CFU/mL ± standard deviation. Different lowercase letters in the columns indicate significant differences (*p* < 0.05) between processes with the same flow rate (T1 and T3, see Table 1), but different doses of UVC. Different capital letters indicate significant differences (*p* < 0.05) between processes with different flowrate, but the same dose of UVC.

Figure 5 shows that in skim milk, single UHPH treatments applied at 200 MPa hardly affected the viability of the *B. subtilis* spores inoculated. When it was combined with UVC treatments lethality increased significantly. In contrast, single UHPH treatments, especially when they were combined with T3 UVC treatments, but lethalities were significantly (*p* < 0.05) lower than the obtained when the UVC treatments were used alone.

### 3.3. Mathematical Modeling of Treatments

Because of the scarce, and even negative effect of UHPH treatments, the mathematical modelling was focused on UVC treatments. Table 3 shows the mathematical models obtained according to the lethality of *B. subtilis* spores for both whole and skim milk. The response variable and different explanatory variables were combined in order to find the model where the predicted values were as close as possible to the observed data. In order to carry the choice of the best model, different statistical parameters have been taken into consideration, prioritizing: (1) the adjusted R^2^ that should be as close as possible to 1; (2) the square root of the mean square error (RSME), that should be as low as possible, and finally, (3) the model where the number of variables is the most reduced. According to that, the best model was the Model 3 in both whole and skim milk. This model was a quadratic approach that takes into consideration NET and log C as variables. The most significant weight in this model was given by the NET value, if the log C variable is not taken into account. Therefore, the applied dose does not play a determining role in terms of the lethality achieved in the different UVC treatments. This fact confirms that increasing the NET increases the chances for one spore to get into the effective area of the reactor, increasing the time that this spore is exposed to a lethal dose.

### 3.4. Kinetics of Inactivation and Estimation of the D_uvc_ Value for the Different UVC Treatments

The adjustments of the data to the inactivation kinetic models included in the GInaFIT tool are shown in Table 4. As previously described, the root mean square error (RMSE) and the R^2^ value were used to determine the fitting accuracy.

In the UVC treatments the calculation of the D value can be of great importance for industries that intend to use this technology to determine the necessary UVC dose to inactivate a specific amount of microorganisms, but in most cases the kinetics of microbial inactivation does not correspond to a linear model and therefore, the calculation of the D value or the time required to reduce 90% of the microbial population (a logarithmic unit) is difficult to estimate. In consequence, it is of great importance to apply the appropriate kinetics in each case and to use the models that better adjust to the real data so as not to overestimate the inactivation capacity of the treatment.

**Figure 6:**
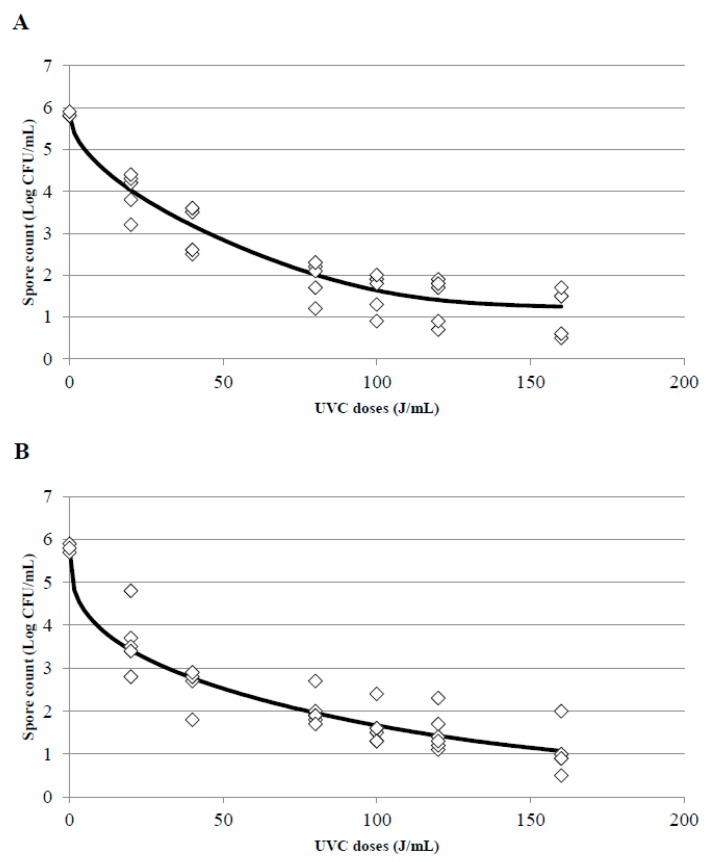
Surviving spores of *B. subtilis* inoculated in whole milk after T2 (**A**) and T3 (**B**) treatments at different UVC doses and adjustment of the data to the Weibull with tail inactivation model using the tool GInaFIT. (◊) indicates the experimental data and the black line the estimated curve once adjusted to the model.

**Figure 7:**
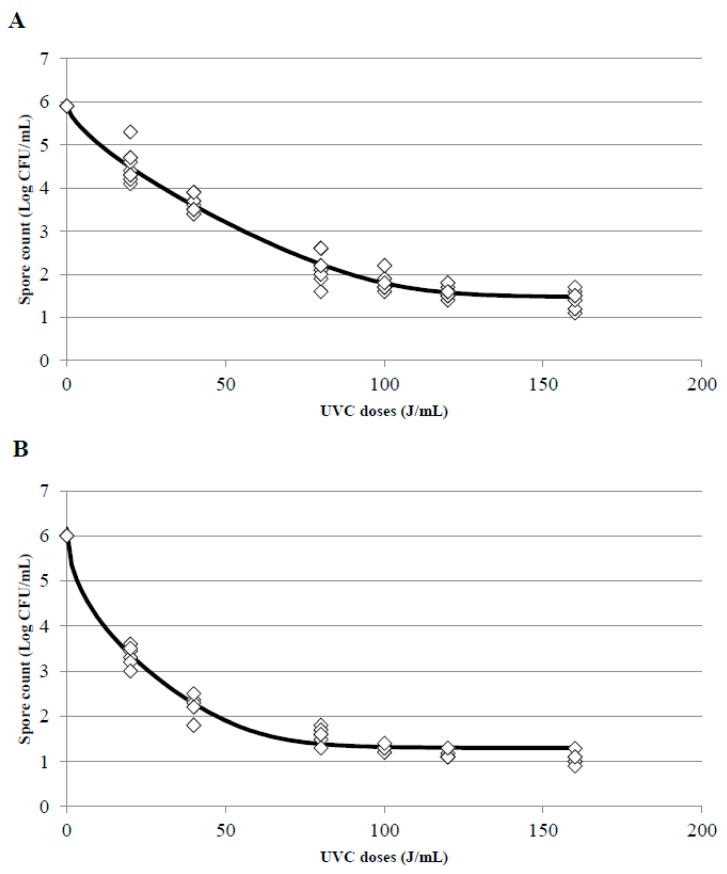
Surviving spores of *B. subtilis* inoculated in skim milk after T2 (**A**) and T3 (**B**) treatments at different UVC doses and adjustment of the data to the Weibull with tail inactivation model using the tool GInaFIT. (◊) indicates the experimental data and the black line the estimated curve once adjusted to the model.

One of the main impediments to adjust experimental data to a linear model is the appearance of tails or shoulders in the inactivation curves; the presence of shoulders in the curves indicates that, at the beginning of treatment, a decrease in the treated microorganism is not observed despite an increase of the doses. To the contrary, the appearance of tails indicates that although the UVC dose increases, a more resistant population will always be observed. In this survey, when the UVC treatments were adjusted, the appearance of tails was the main problem found there, particularly in treatments T2 and T3. Whole and skim milk showed the best adjustment to a Weibull model with a tail (Figure 6A,B for T2 and T3 treatments in whole milk and Figure 7A,B for T2 and T3 treatments in skim milk, respectively). This phenomenon indicates that from a dose of 80 J/mL there is a slowdown in the speed of inactivation of *B. subtilis* spores, and in consequence, when a higher doses (100, 120, or 160 J/mL) were applied spores did not get inactivated at the same inactivation rate than at the lower doses and consequently the inactivation constant were different. The inactivation constant (k) can be used to assess the susceptibility of a microorganism to UVC radiation, and it could be a good indicator as long as the kinetic of inactivation of the microorganism is linear and with only one inactivation constant. Due to the appearance of tails or shoulders, in most cases the inactivation kinetics consisted of two inactivation constants.

Table 4 also shows the 4D value calculated by the GInaFit software in the treatments and models were it was possible. In T1 treatments, the accuracy of the adjustment was not good for any inactivation model and 4D values were not provided. 4D values in T2 and T3 treatments were provided by the Weibull with tail and Biphasic Log-Linear models. In T3 treatments with skim milk the 4D value was also estimated adjusting to the Log-Linear with tail model. Nevertheless, the best adjustment was for the Weibull and Biphasic models.

## 4. Discussion

In matrices with low absorption coefficient different studies were conducted with *B. subtilis* spores, a microorganism commonly used as a control organism in bioassays performed in water treated with UVC due to its moderate resistance and great consistency to UVC inactivation. Chang et al. [39] required approximately 36 mJ/cm^2^ to reduce one log the initial load of *B. subtilis* in water while Sommer et al. [40] achieved this goal with a dose of 20–22 mJ/cm^2^ in a liquid food model with an α(254) of 0.42 cm^−1^. Zhang et al. [41] obtained an inactivation of 0.81 log when treated water with a dose of 78 J/mL. Reverter-Carrión et al. [33] obtained a reduction of 5 log CFU/mL of spores of *B. subtilis* in PBS adjusted to an α(254) of 26 cm^−1^ with caramel after applying a dose of 7.5 J/mL using the same reactor and the same strain of *B. subtilis* that the present study, where the maximum lethality achieved with a single pass was slightly higher than 1 log CFU/mL after a dose of 160 J/mL, due to the greater α(254) of milk.

In whole and skim raw cow’s milk, Choudhary et al. [18] studied the efficiency of UVC treatments on *Bacillus cereus* applying a dose of 11.187 mJ/cm^2^ in a reactor with an inner diameter of 1.6 mm and a flow with a Re number of 713, obtaining a reduction of 2.65 log CFU/mL in whole milk and 1.78 log in skim milk. These lethalities were similar to those obtained in the present study with a T3 treatment at a dose of 40 J/mL (4165.6 mJ/cm^2^) and 20 J/mL (2082.8 mJ/cm^2^) in whole and skim milk, respectively. As in the study of Choudhary et al. [18], in the present survey the efficiency of UVC treatments was higher in skim milk than in whole milk at small doses (20 J/mL and 40 J/mL), probably due to the α(254) of both types of milk and its turbidity value. However, these α(254) (220 cm^−1^ and 170 cm^−1^ for whole and skim milk, respectively) were lower than the observed ones in the milk samples used in this study (801 cm^−1^ and 264 cm^−1^ for whole and skim milk, respectively). This difference may be because, in this study, commercial milk was used, which in addition to the UHT treatment had suffered a homogenization treatment. The greater effectiveness observed in skim milk is also explained because of the estimated distance at which *B. subtilis* spores receive the most effective UVC radiation is higher than in whole milk, as can be seen in Table 2, and then when NET increases so do the probability of the presence of the spores within this zone.

Regarding the effect of the application of UVC treatments in other matrices of high α(254), Bandla et al. [42] evaluated the effect of a dose of 11,187 mJ/cm^2^ on *Bacillus cereus* spores in a soybean drink with an α(254) of 163 cm^−1^, lower than that reported in the milks used in this study. The reactor used was a spiral tube with two different internal diameters (1.6 mm and 3.2 mm), generating two values of Re number that were higher than those achieved in this study (Table 1). Due to that, they reported reductions of 3.22 and 1.66 log CFU/mL with the smallest and largest diameter, respectively. In this study, reductions of 1.47 log were reported for a dose of 20 J/mL equivalent to 2,082.8 mJ/cm^2^ in the T3 treatment and 4 log CFU/mL for a dose of 80 J/mL (equivalent to 8,323 mJ/cm^2^).

These results indicate that in matrices with a high absorption coefficient, the Re number effect influences the lethality achieved. This result can be explained by the “boundary layer theory” proposed by Prandtl (1925) [43], whereby a moving fluid tends to lose speed when it is in contact with a small adjacent layer of solid due to friction losses. This phenomenon is what would happen in the reactor when the flowing matrix is in contact with the quartz that protects the lamp, losing speed and thus decreasing the Re number, including the possibility of creating different layers that would not change its depth when passes throughout the reactor. This causes that in matrices of high α(254), the layer closest to the source of emission of UVC radiation is overexposed, but the inner layers do not receive a sufficient lethal dose. In this type of matrix, to increase the number of passes through the reactor (NET) increases, therefore, the probability that a particular particle is in the area of highest exposure to UVC radiation, increasing the lethal effect. Therefore, in matrices of high absorption coefficient, it seems more effective to increase the number of passes or NET value than to increase the Re value in order to obtain greater reductions.

The high resistance of bacterial spores to UHPH treatments has already been described in milk by Amador-Espejo et al. [32], observing that *B. subtilis* has a higher resistance to these treatments than the spores of other bacteria. In order to achieve significant reductions of at least 5 log of this microorganism, it was necessary to increase the pressure to 300 MPa and preheating the milk to a temperature of at least 80 °C to increase the thermal effect. Thus, to combine UHPH with another technology or unfavourable condition seems interesting in order to increase lethality and achieve significant reductions of bacterial spores in foods. In this study it has been assessed whether the combination of UHPH with UVC could be an option to increase the effectiveness of both treatments, reducing the number of passes needed in the case of UVC, and the need to increase the temperature in the case of UHPH, which *a priori* would less affect the properties of the matrix. However, in this study, there was no clear synergistic or additive effect between both technologies. In fact, in some cases, lethalities obtained on *B. subtilis* spores after applying combined treatments, especially in skim milk, decreased concerning single UVC treatments at the same doses. These results could be explained by the modifications caused by UHPH on the structures of fat globules and casein micelles [44]. Table 5 shows the effect of UHPH on the absorption coefficient and the turbidity of milks used in this study, showing that UHPH treatments increase the turbidity and the α(254) values in both whole and skim milk. The changes suffered by the matrix may have high relevance in the effectiveness of the combination of technologies since in a previous study Reverter-Carrión et al. [33] observed an additive effect when both technologies were applied over the same microorganism but inoculated in a buffered solution (PBS) without the interference of suspended particles.

Table 4 shows the estimated 4D_uvc_ values for *B. subtilis* in whole and skim milk once the inactivation curve was fit to a Weibull with tail model. The most effective treatment in whole milk showed to be the T3, being necessary a dose of 91.2 J/mL to inactivate 4 log CFU/mL of spores, but the difference with the T2 treatments was small. On the contrary, T3 treatments showed to be much more efficient in skim milk, where a dose of 41.6 J/mL would be enough to achieve this goal. No previous references were found of D_uvc_ values for *B. subtilis* or any other sporulated bacteria in milk. Crook et al. [2] reported a D_uvc_ value of 0.73 J/mL for *L. monocytogenes* in milk, and of 0.556 J/mL for *E. coli*, but these bacteria are much more sensitive to UVC than *B. subtilis* [9]. Concerning other matrices, Nicholson et al. [45] calculated the D_uvc_ value for spores of *B. subtilis* in phosphate-buffered saline solution, estimating it in 120 J/m^2^, what implies a dose of 480 J/m^2^ to achieve a 4D reduction. In the present survey, for the most effective treatment (T3), the estimated 4D_uvc_ would be above 43,300 J/m^2^. In that case, the least interfering matrix used there would justify the differences.

## 5. Conclusions

Recirculation seems to be a good strategy in the application of UVC treatments to matrices with a high degree of interference, such as milk, since the mixing effect ensures that microbial spores (or planktonic cells) will be in the area of maximum efficacy (where the action of UVC radiation is the highest) enough time to be inactivated. This hypothesis seems to be demonstrated by the fact that NET was the most determining variable in order to predict the lethality caused over the spores of *B. subtilis*. However, the possibility of weakening the resistance of the spores of *B. subtilis* by applying another physical treatment in advance, such as UHPH, proved to be ineffective in this case, despite the favorable references observed previously [33,46], probably due to the effect of UHPH on the matrix that increases the protective effect on spores. It seems that achieving the goal of a commercial sterilization of milk by means of UVC is complicated since the estimated 4D_uvc_ values of the most effective treatments are too high.

## Figures and Tables

**Table 1:** Parameters of the UVC Treatments Tested.

Treatment	Flow Rate (mL/s)	NET	RPM	Retention Time (s)	UVC Dose (J/mL)	Reynolds Number
T1	≤2.9	2	21	24	20	52
			12	48	40	26
			6	96	80	13
			5	120	100	10
			3	144	120	9
			1.5	192	160	7
T2	41.9	30	300	360	20	658
		60		720	40	
		121		1440	80	
		151		1800	100	
		181		2160	120	
		241		2880	160	
T3	64.6	47	500	360	20	1015
		93		720	40	
		186		1440	80	
		233		1800	100	
		279		2160	120	
		372		2880	160	

NET: number of entries to the tunnel; RPM: revolutions per minute (RPM).

**Table 2:** Calculation of the effective distance, expressed in mm where the dose received is close to 1 mJ/cm^2^.

Matrix	Doses (mJ/cm^2^)	Effective Distance (mm)
Whole milk	1.034	0.02
	0.861	0.02
Skim milk	1.005	0.06
	0.946	0.06

**Table 3:** Mathematical models depending on the lethality UVC treatments on the *B. subtilis* spores obtained in whole and skim milk.

	Models	*R* ^2^	RMSE
**Whole milk**			
**1**	$\mathrm{Lethality}=3.56-0.71\times {\;\mathrm{Log}}_{C}+0.015\times \mathrm{UVC}+0.05\;\times \;R$	0.83	0.70
**2**	$\mathrm{Lethality}=19.17-3.02\;\times {\;\mathrm{Log}}_{C}+0.012\times \mathrm{NET}$	0.83	0.69
**3**	$\mathrm{Lethality}=6.557-1.002\;\times {\;\mathrm{Log}}_{C}+0.029\;\times \;\mathrm{NET}-0.00005\;\times {\;\mathrm{NET}}^{2}$	0.94	0.40
**4**	$\mathrm{Lethality}=4.86-0.77\;\times {\;\mathrm{Log}}_{C}+0.0048\;\times \;\mathrm{UVC}+0.029\times \mathrm{FR}-0.0078\;\times \;\mathrm{NET}$	0.86	0.63
**5**	$\mathrm{Lethality}=2.696-0.39\;\times {\;\mathrm{Log}}_{C}+0.0012\;\times \;\mathrm{UVC}+0.009\;\times \;\mathrm{FR}+0.027\;\times \;\mathrm{NET}-0.00005\;\times {\;\mathrm{NET}}^{2}$	0.94	0.40
**Skim milk**			
**1**	$\mathrm{Lethality}=-14.74+2.79\;\times {\;\mathrm{Log}}_{C}+0.014\;\times \;\mathrm{UVC}+0.017\;\times \;\mathrm{FR}$	0.91	0.52
**2**	$\mathrm{Letalidad}=-10.1+2.12\;\times {\;\mathrm{Log}}_{C}+0.0086\;\times \;\mathrm{NET}$	0.90	0.53
**3**	$\mathrm{Lethality}=-4.2+0.97\;\times {\;\mathrm{Log}}_{C}+0.023\;\times \;\mathrm{NET}-0.00004\;\times {\;\mathrm{NET}}^{2}$	0.95	0.36
**4**	$\mathrm{Lethality}=-12.53+2.47\;\times {\;\mathrm{Log}}_{C}+0.0077\;\times \;\mathrm{UVC}+0.0072\;\times \;\mathrm{FR}+0.00472\;\times \;\mathrm{NET}$	0.92	0.48
**5**	$\mathrm{Lethality}=-4.367-0.92\;\times {\;\mathrm{Log}}_{C}+0.0049\;\times \;\mathrm{UVC}+0.01\;\times \;\mathrm{FR}+0.02\;\times \;\mathrm{NET}-0.00004\;\times {\;\mathrm{NET}}^{2}$	0.96	0.34

Log C: quantity of inoculated microorganisms expressed as log CFU/mL; UVC, the dose of UVC radiation, expressed in J/mL; FR: treatment flow rate, expressed in mL/s; NET: number of entries to the tunnel.

**Table 4:** Adjustment to kinetic model and estimation of the corresponding 4D_uvc_ value (Four decimal reduction value: UVC dose necessary to reduce 4 log CFU/mL) of *B. subtilis* spores in whole and skim milk according to the UVC treatments applied using the GInaFIT tool.

Treatment	Type of Milk	Inactivation Model	RMSE	*R* ^2^	4D_uvc_ (J/mL)
**T1**	Skim	Log-Linear Regression	0.2433	0.592	-
		Log-Linear with tail	0.2269	0.654	-
		Weibull	0.2087	0.707	-
		Biphasic	0.2101	0.710	-
	Whole	Log-Linear Regression	0.1143	0.242	-
		Log-Linear with tail	0.2331	0.202	-
		Weibull	0.2318	0.210	-
		Biphasic	0.2330	0.224	-
**T2**	Skim	Log-Linear Regression	0.7050	0.769	-
		Log-Linear with tail	0.5092	0.883	-
		Weibull with tail	0.4491	0.911	89.6
		Biphasic	0.4749	0.901	116.8
	Whole	Log-Linear Regression	0.5441	0.839	-
		Log-Linear with tail	0.2731	0.960	-
		Weibull with tail	0.2375	0.970	94.4
		Biphasic	0.0646	0.966	126.4
**T3**	Skim	Log-Linear Regression	0.6673	0.811	-
		Log-Linear with tail	0.3124	0.952	44.8
		Weibull with tail	0.2294	0.975	43.2
		Biphasic	0.2059	0.980	41.6
	Whole	Log-Linear Regression	0.7465	0.705	-
		Log-Linear with tail	0.3263	0.571	-
		Weibull with tail	0.4637	0.892	91.2
		Biphasic	0.2157	0.464	97.6

**Table 5:** Effect of ultra-high pressure homogenization (UHPH) treatments on the absorption coefficient and the turbidity of whole and skim milk.

Optical Parameter	Skim Milk		Whole Milk	
	Before UHPH	After UHPH	Before UHPH	After UHPH
α(254) (cm^−1^)	264 ± 0.03	412 ± 0.02	801 ± 0.02	1012 ± 0.02
Turbidity (NTU)	18,416 ± 0.04	21,630 ± 0.03	77,967 ± 0.01	102,458 ± 0.01
